# Giant ascending aortic aneurysm with impending rupture as presentation of cutis laxa 1B: a case report

**DOI:** 10.1093/ehjcr/ytad530

**Published:** 2023-11-20

**Authors:** Alejandro Used-Gavín, José María Larrañaga-Moreira, Rafael Lago-Cascudo, Víctor X Mosquera-Rodríguez, Roberto Barriales-Villa

**Affiliations:** Inherited Cardiovascular Diseases Unit, Hospital Universitario A Coruña (HUAC), As Xubias 84, 8th floor, 15006 A Coruña, Spain; Instituto de Investigación Biomédica de A Coruña (INIBIC), Universidade da Coruña (UDC), Servizo Galego de Saúde (SERGAS), As Xubias 84, 15006 A Coruña, Spain; Inherited Cardiovascular Diseases Unit, Hospital Universitario A Coruña (HUAC), As Xubias 84, 8th floor, 15006 A Coruña, Spain; Instituto de Investigación Biomédica de A Coruña (INIBIC), Universidade da Coruña (UDC), Servizo Galego de Saúde (SERGAS), As Xubias 84, 15006 A Coruña, Spain; Instituto de Investigación Biomédica de A Coruña (INIBIC), Universidade da Coruña (UDC), Servizo Galego de Saúde (SERGAS), As Xubias 84, 15006 A Coruña, Spain; Pathology Department, Hospital Universitario A Coruña (HUAC), A Coruña, Spain; Instituto de Investigación Biomédica de A Coruña (INIBIC), Universidade da Coruña (UDC), Servizo Galego de Saúde (SERGAS), As Xubias 84, 15006 A Coruña, Spain; Cardiac Surgery Department, Hospital Universitario A Coruña (HUAC), As Xubias 84, 8th floor, 15006 A Coruña, Spain; Inherited Cardiovascular Diseases Unit, Hospital Universitario A Coruña (HUAC), As Xubias 84, 8th floor, 15006 A Coruña, Spain; Instituto de Investigación Biomédica de A Coruña (INIBIC), Universidade da Coruña (UDC), Servizo Galego de Saúde (SERGAS), As Xubias 84, 15006 A Coruña, Spain; Centro de Investigación Biomédica en Red (CIBERCV), Av. Monforte de Lemos, 3-5, 28029 Madrid, España

**Keywords:** Cutis laxa, Aortic aneurysm, Genetics, *EFEMP2*, Case report

## Abstract

**Background:**

Thoracic aortic aneurysms are rarely symptomatic but can result in acute aortic syndromes, associated with a high mortality rate. While most cases may be acquired, a genetic basis is evident in approximately 20–25% of the cases, especially among patients under 50 years of age, and those exhibiting syndromic features or family history. Although autosomal dominant inheritance is predominant in familial aortopathies, exceptions exist, such as cutis laxa 1B (CL1B)-related aortic disease, caused by variants in *EFEMP2* gene, that follows an autosomal recessive inheritance pattern.

**Case summary:**

We present the case of a 26-year-old male with a giant ascending aorta aneurysm and massive pericardial effusion, which was ultimately diagnosed of CL1B due to the p.Ser137Cys variant in the *EFEMP2* gene in homozygosis. The patient underwent successful ascending aorta replacement (Bentall´s procedure). There were not complications or further events after 2 years of follow-up.

**Discussion:**

This case underscores the importance of genetic testing in young patients presenting with aortopathies, syndromic features, or atypical presentations, irrespective of family history.

**Learning points**

- Autosomal recessive cutis laxa 1B is a disorder linked to variants in the *EFEMP2* gene.
- The presence of large ascending aortic aneurysms at young ages and accompany extravascular features should prompt suspicion of this condition.
- Genetic testing is warranted in individuals with aortopathies presenting at young ages, regardless of their familiar history.

## Introduction

Thoracic aortic aneurysms are rarely symptomatic, but they can show progressive growth, producing symptoms caused by the compression of adjacent structures.^1^ They may result in acute aortic syndromes, which are associated with high mortality rates. While thoracic aortic disease (TAD) can be acquired, at least 20–25% have a genetic basis, especially in patients under 50 years of age and those with syndromic features or a family history.^2^

Inherited aortopathies can be syndromic. In such cases, the involved genes are components of the extracellular matrix (e.g. *FBN1*, Marfan syndrome) or part of the TGF-beta pathway (e.g. Loeys–Dietz syndrome). Non-syndromic cases are mainly associated with bicuspid aortic valve or genes related to vascular smooth muscle (e.g. *ACTA2*).^2,3^

Autosomal dominant inheritance is predominant in familial aortopathies. However, there are exceptions, such as cutis laxa 1B (CL1B)-related aortic disease. This disease primarily exhibits a vascular phenotype and is caused by pathogenic variants in the *EFEMP2* gene, with an autosomal recessive inheritance pattern.^4–6^ In this context, we present a patient with a giant ascending aortic aneurysm and massive pericardial effusion. Ultimately, he was diagnosed with CL1B due to an homozygous variant (p.Ser137Cys) in the *EFEMP2* gene.

## Summary figure

Timeline and brief of the case report. AR, aortic regurgitation; CT, computed tomography; *EFEMP2*, EGF containing fibulin extracellular matrix protein 2; ICU, intensive care unit; TEE, transoesophageal echocardiogram; TTE, transthoracic echocardiogram.

**Figure:**
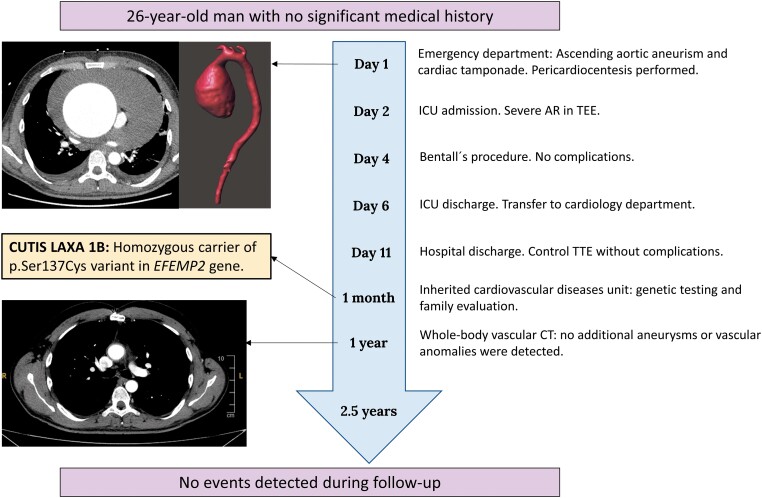


## Case presentation

**Figure 1:**
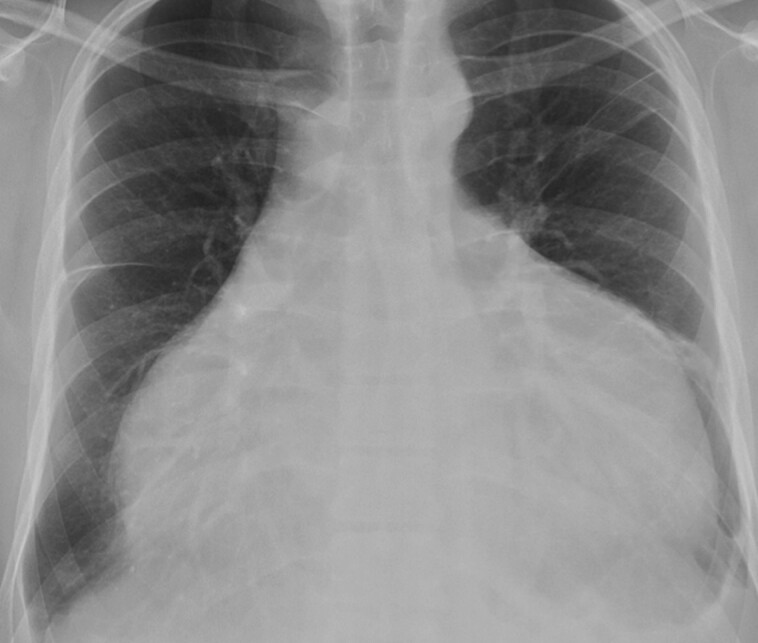
Posteroanterior chest X-ray, note the significantly enlarged cardiac silhouette.

**Figure 2:**
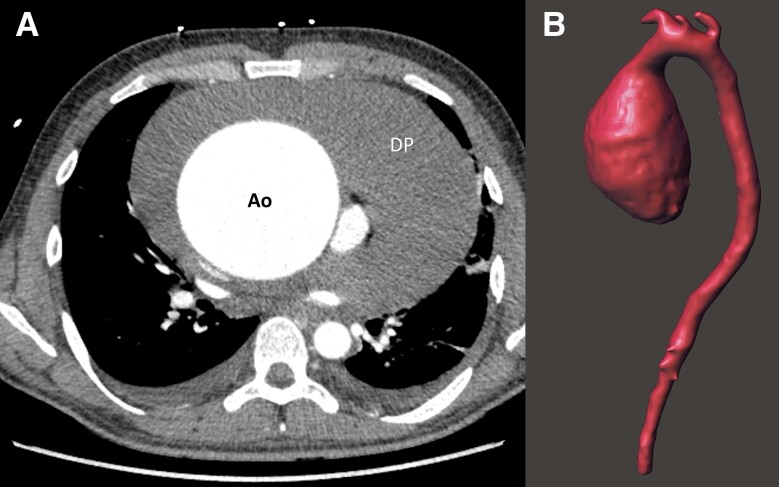
(*A*) Contrast-enhanced axial computed tomography (CT) reveals an aneurysmal ascending aorta (ao) along with a severe pericardial effusion (PE). (*B*) Isovolumetric reconstruction of the entire aorta displays the distinctive ‘sausage-like’ morphology of the ascending aortic aneurysm, while the remainder of the aorta appears to be of normal size.

A 26-year-old Colombian male without past medical history presented to the emergency department with a 1-month history of dry cough, progressive dyspnoea, and orthopnoea. On physical examination, his blood pressure was normal (110/70 mmHg), but he exhibited tachycardia (110 b.p.m.), and elevated jugular venous pressure. No cardiac murmurs or lung rales were detected. The electrocardiogram revealed generalized low voltages, and blood test results were unremarkable. A chest X-ray showed significantly enlarged cardiac silhouette (*Figure 1*). A subsequent computed tomography (CT) scan (*Figure 2*) confirmed the presence of a giant aneurysm in the ascending thoracic aorta (maximum diameter 96 mm) and a massive pericardial effusion (maximum diameter 65 mm). The remaining aorta and its branches appeared to be of normal calibre. A bedside echocardiogram indicated signs of cardiac tamponade and, because of rapid worsening symptoms, emergent pericardiocentesis was performed to stabilize the patient. The obtained pericardial fluid was serosanguinous. Subsequently, the patient was admitted to the intensive care unit for further stabilization.

**Figure 3:**
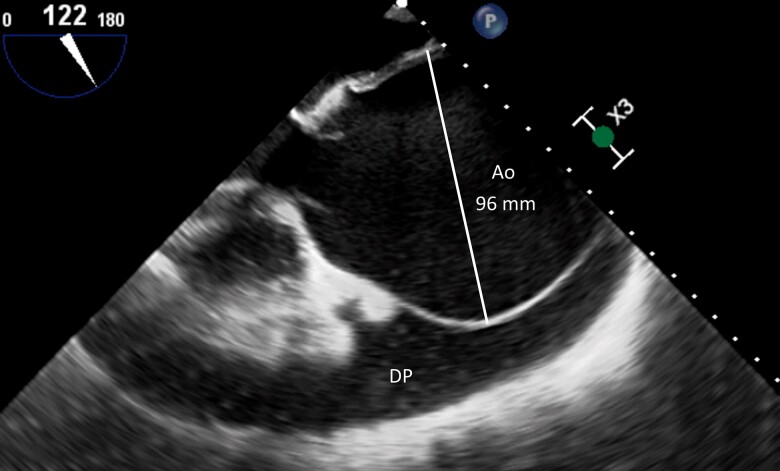
Post-pericardiocentesis transoesophageal echocardiogram in the mid-oesophageal view showing a large ascending aortic aneurysm (Ao) and residual pericardial effusion (PE).

**Figure 4:**
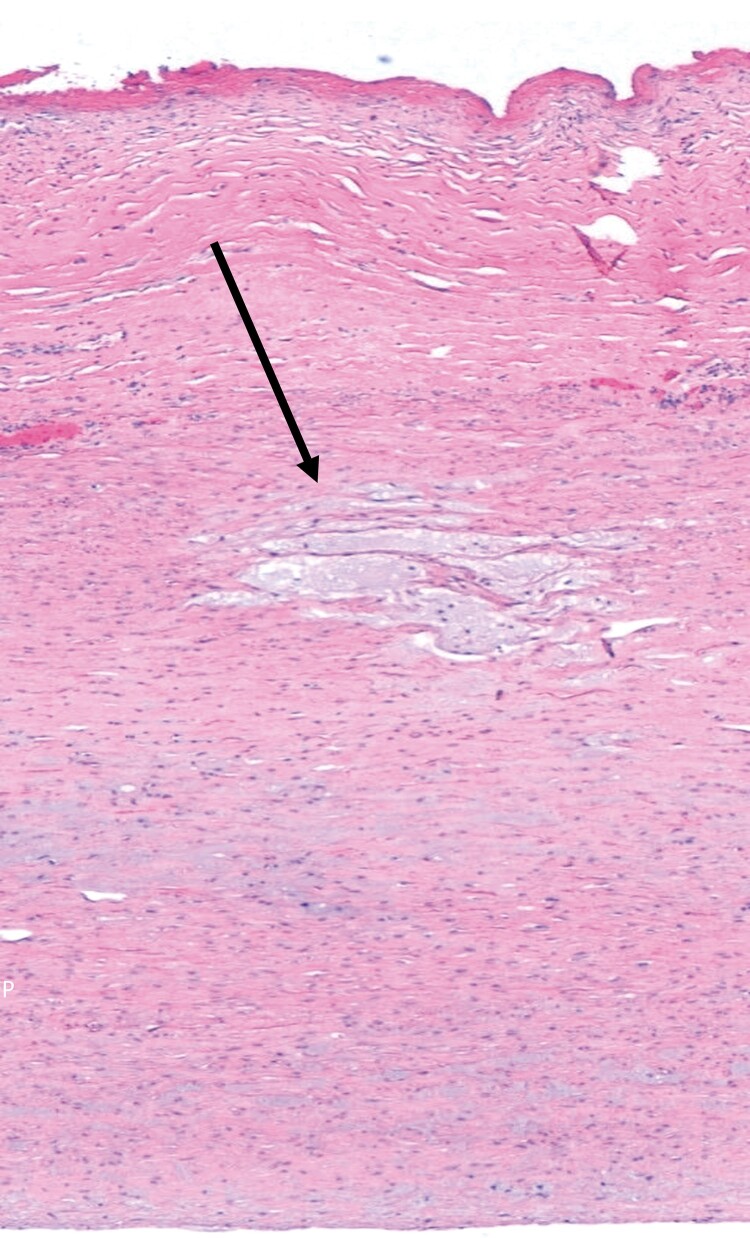
Histopathological examination of the resected aorta with hematoxylin–eosin staining revealed disorganized elastic fibres in tunica media (arrow).

A transoesophageal echocardiogram revealed severe aortic regurgitation (*Figure 3*), with a broad and dense central jet resulting from a large coaptation defect secondary to the enlarged ascending aorta. Holodiastolic flow reversal in the descending aorta was observed, with an end-diastolic velocity of 40 cm/s. The estimated effective regurgitant orifice area was 1.5 cm^2^. Left ventricular diameters and function were normal, suggesting an acute/sub-acute onset. The overall clinical presentation strongly indicated an impending aortic rupture. On 4th day, the patient underwent ascending aorta replacement with a Bentall´s procedure using a 25 mm Carboseal^TM^ mechanical prosthesis and a 28 mm Dacron aortic graft. The excised tissue was sent for pathological analysis, which revealed a thin aortic wall and histological evidence of medial degeneration and disorganization of elastic fibres (*Figure 4*). There were not intra- nor post-operative complications, and the patient was discharged from the hospital after 1 week.

Following discharge, the patient was assessed by the Inherited Cardiovascular Diseases Unit. While there was no significant family history, physical examination unveiled dolichocephaly with mild retrognathia, arachnodactyly, joint laxity, and a high-arched palate. The Gante’s systemic score was 4. A genetic study using next generation sequencing (NGS) encompassing 64 genes related to aortic and connective tissue disorders was conducted. The genetic variant c.409A > T, p.Ser137Cys, in *EFEMP2* was identified in homozygous state. Cascade screening of first-degree relatives revealed that they were healthy heterozygous carriers.

Subsequent CT scan of the entire vascular system 1 year after surgery did not reveal aneurysms in other locations, although arterial tortuosity was noticed. After 2 years of follow-up, the patient has been managed with losartan 50 mg and acenocoumarol, with no further reported events.

## Discussion

Family history and genetic testing are crucial components of the diagnostic work-up for patients with TAD. Genetic testing, utilizing a validated NGS panel, is recommended for individuals exhibiting syndromic features, family history of TAD, or presenting at an age younger than 60 years. A positive result has diagnostic and prognostic implications for the patient, and cascade testing should be conducted among at-risk relatives with the same purpose.^1,2^ In recent years, several new candidate genes have been linked to TAD, although validation efforts have demonstrated robust evidence for only a few genes, with *EFEMP2* having modest evidence.^7^

The *EFEMP2* gene encodes fibulin-4, a crucial protein involved in the development and maintenance of the extracellular matrix and its elastic fibres. Fibulin-4 interacts with other proteins such as fibrillin-1, lysyl-oxidase, and tropoelastin. Additionally, it plays a role in the differentiation of smooth muscle cells and is involved in the TGF-beta pathway.^4,8^ Fibulin-4 expression is particularly prominent in conductance arteries, notably in the ascending thoracic aorta.^9^

**Figure 5:**
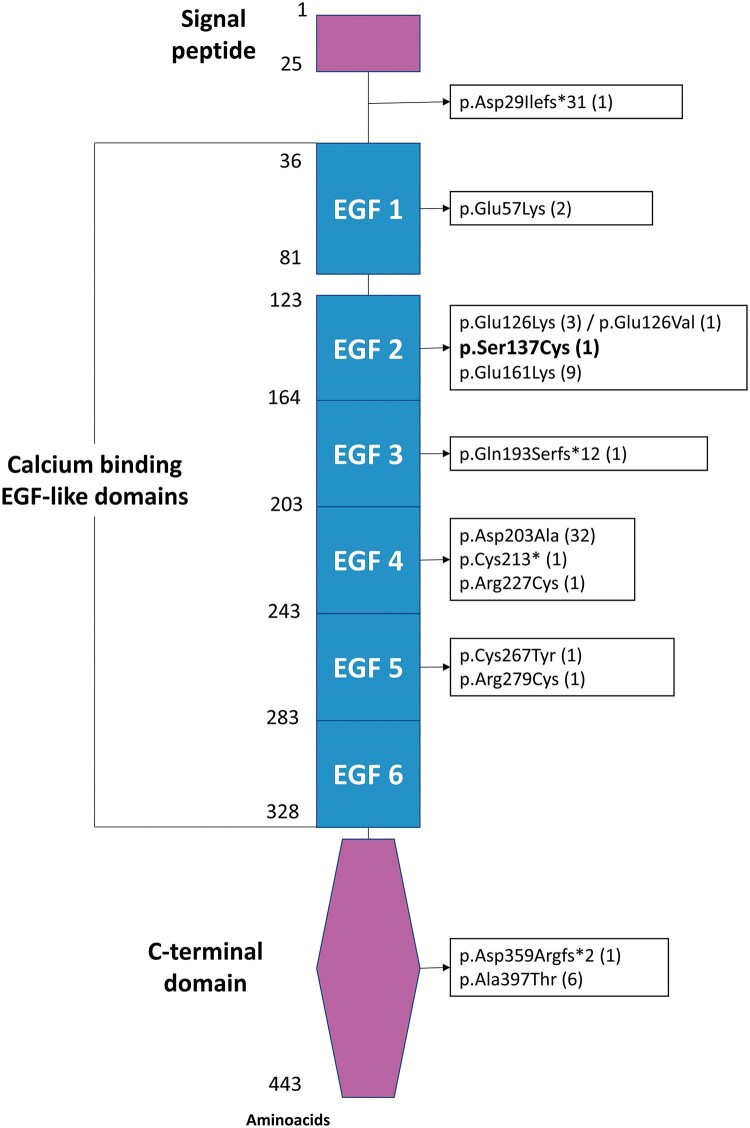
The structure of the *EFEMP2* gene and previously reported variants are shown, with our patient’s variant highlighted in bold. (*n*), number of carriers of each variant; EGF, epidermal growth factor.

The *EFEMP2* gene comprises six epidermal growth factor-like (EGF-like) domains and a C-terminal domain. Biallelic pathogenic variants in *EFEMP2* cause CL1B, a rare disease characterized by reduced fibulin-4 levels. This reduction leads to elastin disorganization and a secondary increase in TGF-beta signalling.^8^ There are at least 45 cases and 12 distinct pathogenic variants described in the literature (*Figure 5*).^4–6,8,10–15^ The most common variant is p.Asp203Ala, which has a founder effect in India.^6^ Truncating variants result in a complete lack of fibulin-4, resulting in a severe phenotype with skeletal involvement often leading to perinatal death.^8,12^ Missense variants impact specific calcium or cysteine binding residues critical for fibulin-4 stabilization, resulting in a variable deficiency and milder phenotypes, characterized by predominant vascular involvement, like our case.^5,6,8,10,13–15^ Reported cases typically present during childhood, characterized by large ascending aortic aneurysms (sometimes referred to as ‘sausage’ aorta^11,15^), that frequently cause symptoms due to compression of nearby structures. Severe pericardial effusion has been observed, while aortic dissection is infrequent.^13^ The aortic isthmus tends to have normal or hypoplastic dimensions, and tortuosity and aneurysms can be found in the descending aorta and pulmonary artery.^6,8,11,15^ The extravascular phenotype varies depending on the severity of fibulin-4 deficiency, often including mild skin and joint laxity, arachnodactyly, high palate or hypertelorism, resembling another connective tissue diseases.^4,8,10,13^ More severe phenotype with skeletal involvement is also described in cases of missense variants causing profound fibulin-4 deficiency.^10^ Other manifestations like emphysema, diaphragmatic abnormalities, hernias or keratoglobus have also been reported.^4,10^

There is lack of long-term follow-up data for CL1B patients, and imaging surveillance recommendations are absent. In our view, itis reasonable to consider whole-body vascular imaging, ideally performing magnetic resonance imaging, initially on an annual basis. Then, if stability is established, every 2 years.^4^

The p.Ser137Cys variant explains our patient’s phenotype. This variant appears in the Latino control population with low frequency (22/34 566 alleles; gnomAD) and has previously been reported in a 3-year-old patient with a giant aortic aneurysm and no evident extravascular phenotype.^14^ This variant affects a highly conserved residue, and multiple bioinformatic predictors project a deleterious effect. Considering these factors, it is classified as likely pathogenic.

Our case, featuring the oldest patient diagnosed with CL1B, underscores the importance of genetic testing in young individuals with aortopathies, syndromic features, or atypical presentation, irrespective of family history.

## Data Availability

All data are incorporated into the article.

## Lead author biography

Alejandro Used-Gavín graduated in 2017 at Universidad de Navarra, Pamplona, Spain. He is currently a Cardiology Resident at CHU A Coruña, Spain.

## Notes

**Consent:** The authors confirm that written consent for the submission and publication of this case report, including images and associated text has been obtained from the patient in line with COPE guidelines.

**Funding:** None.
